# The Health of School Girls

**Published:** 1897-02-13

**Authors:** 


### EDITOR'S LETTER-BOX.

[Our Correspondents are reminded that prolixity is a great bar to publication, and that brevity of style and conciseness of statement greatly facilitate early insertion.]

### THE HEALTH OF SCHOOL GIRLS.

Dr. John Moir, St. Andrew's, Fife, writes: In the "System of Gynæcology by Many Writers," lately published by Macmillan and Co., and recently reviewed in The Hospital, Dr. W. S. Playfair, one of the editors, contributes a paper on the "Nervous System in Relation to Gynæcology," a subject on which he, from special study and large experience, can speak with authority—authority which will be accepted by the profession and laity almost without reserve. On this very account may I be allowed to call attention to what seems to me to be a somewhat sweeping statement on his part as regards the great schools for girls in this country, a subject important in the highest degree to every parent who has daughters to educate.

Dr. Playfair says: "The one great fault of those who manage these educational establishments is that they have too often started on the absolutely untenable theory that the sexual factor is of secondary importance, and that there is little, if any, real distinction between a girl between the ages of 14 to 20 and a boy of the same age. I know of no large school for girls where the absolute distinction which exists between boys and girls, as regards the dominant menstrual function, is systematically cared for and attended to."

The assertions contained in the above extract and already noticed in your columns prompt me to challenge the accuracy and fairness of his opinion, as within my own limited field of experience I know of at least two schools to which these remarks are quite inapplicable, viz., St. Leonard's School, St. Andrews, and Wycombe Abbey School, High Wycombe. Under the present head mistress of the latter school a system of care as regards health was elaborated during her fourteen years' headship at St. Leonards, which is carefully carried out at both schools, and, to my mind, leaves little to be desired. It is indisputable that great risk is run in ignoring the sexual developmental change in a girl by exacting too much from her mentally, and in allowing her to take at all times unrestricted exercise, but great judgment and tact are required in making this restriction, not to make too much of the condition at a time when the girl is, in any case, off her balance nervously. Dr. Playfair, in another paragraph of the same paper, very properly deplores the concentration of the attention of nervous, emotional women upon certain organs and symptoms by uncalled-for and misdirected treatment. Is there not the same—even a greater—risk in making too much of a strictly physiological function in the young and growing girl, whose nervous stability cannot certainly be as great as—*cæteris paribus*—it would be in after years. I have had the honour of being medical officer to the St. Leonard's School since its foundation—now nearly 20 years ago. From personal knowledge and experience of its working, I can most unhesitatingly say that, notwithstanding the prominent position given to outdoor games and physical education generally, it is carefully inculcated on those who are most directly responsible for the health of the girls to keep note of the special circumstances of the health of each, and a system is in vogue such that no carelessness on a girl's part can escape detection. A veto is thus put on all exercise that, for the time, might be injurious. In every case where there seems need for it the girl is kept quietly resting at certain times, and in exceptional cases confined to bed. Intensely keen though the interest be which attaches to a "house-match," many and many a time have I known a team on the lacrosse ground and cricket field crippled by the enforced absence of one or more of its mainstays. When a girl is noticed to be flagging, either in or out of school, she is sent to the sick house for a few days' rest, and there she is under the care of the lady superintendent—a certificated nurse of wide experience. The school has also the advantage of having as resident director of the gymnasium a lady well qualified by special education and training, who modifies or discontinues exercises in the gymnasium unsuited to the physique or temporary condition of any of her pupils. What would you have more? I might add that careful note is made each term of the growth in height and weight of every girl, and my attention, as medical officer, is at once called to any unsatisfactory condition. This, I am bound to say, is, considering the number of girls in session—two hundred—rare, for, thanks to the excellent school system as regards hours of study, the attention paid to physical exercise, and the bracing and invigorating surroundings, a race of girls has been educated at St. Leonard's School that has developed, and is developing, into types of healthy English womanhood.

